# Membrane Protein Identification in Rodent Brain Tissue Samples and Acute Brain Slices

**DOI:** 10.3390/cells8050423

**Published:** 2019-05-08

**Authors:** Sarah Joost, Stefan Mikkat, Michael Wille, Antje Schümann, Oliver Schmitt

**Affiliations:** 1 Institute of Anatomy, University Medical Center Rostock, 18057 Rostock, Germany; Sarah.Joost@med.uni-rostock.de (S.J.); michael-wille@gmx.de (M.W.); antje.schuemann@uni-rostock.de (A.S.); 2 Core Facility Proteome Analysis, University Medical Center Rostock, 18057 Rostock, Germany; stefan.mikkat@med.uni-rostock.de

**Keywords:** plasma membrane proteins, liquid chromatography-mass spectrometry, murine acute brain slices, reproducibility, rat cerebellum

## Abstract

Acute brain slices are a sample format for electrophysiology, disease modeling, and organotypic cultures. Proteome analyses based on mass spectrometric measurements are seldom used on acute slices, although they offer high-content protein analyses and explorative approaches. In neuroscience, membrane proteins are of special interest for proteome-based analysis as they are necessary for metabolic, electrical, and signaling functions, including myelin maintenance and regeneration. A previously published protocol for the enrichment of plasma membrane proteins based on aqueous two-phase polymer systems followed by mass spectrometric protein identification was adjusted to the small sample size of single acute murine slices from newborn animals and the reproducibility of the results was analyzed. For this, plasma membrane proteins of 12 acute slice samples from six animals were enriched and analyzed by liquid chromatography-mass spectrometry. A total of 1161 proteins were identified, of which 369 were assigned to membranes. Protein abundances showed high reproducibility between samples. The plasma membrane protein separation protocol can be applied to single acute slices despite the low sample size and offers a high yield of identifiable proteins. This is not only the prerequisite for proteome analysis of organotypic slice cultures but also allows for the analysis of small-sized isolated brain regions at the proteome level.

## 1. Introduction

Acute brain slices are an important sample format in neuroscience [1]. The 300–500 µm thick acute slices of rodent brains are the basis, among other things, for organotypic slice cultures [2], electrophysiological applications [3], as well as functional local synaptic circuitry analyses. Organotypic brain slice cultures are an ex vivo model for maintaining the three-dimensional structure of rodent brain tissue in culture over weeks [4]. They can be easily manipulated, and neuronal as well as glial cell types are available for almost all commonly used analytical options in these cultures. An exception is mass spectrometry (MS)-based proteome analyses, which are hardly ever applied to single brain slices, probably due to the small sample mass of one single acute slice or even a subregion of an acute slice. However, MS-based protein identification and quantification allows for the determination of complex quantitative protein profiles for differential or explorative analyses. Only a few studies were performed on the proteome of single slices. Bowling and colleagues [5] analyzed the stimulus-triggered protein synthesis in acute hippocampal slices. In different studies, plasma membrane proteins were extracted by biotinylation and streptavidin-pulldown and subsequently identified by MS in proof-of-principle approaches from slices of the visual cortex [6] and the hippocampus [7].

Plasma membrane proteins turn out to be of special interest in neuroscience as they comprise ion channels, neurotransmitter receptors, ion transporters, and many more sizeable protein classes with particular importance on neuronal functions [8,9]. Furthermore, the myelin sheaths in the central nervous system are formed by the differentiated plasma membrane of a myelinating glial cell, the oligodendrocyte, which is involved in many pathological processes, including immune-mediated destruction or metabolic-induced cell stress. For the specific enrichment of plasma membrane proteins, several methods were reported that are either based on chemical labeling of membrane proteins or on macromolecular and physicochemical properties of the plasma membrane itself [10]. In the first case, different labeling strategies with biotin followed by streptavidin-pulldown are widely employed [11,12]. Particular plasma membrane properties allow for their isolation by differential and density gradient centrifugation [10] and by aqueous polymer two-phase enrichment [13]. This method uses a mixture of dextran and polyethylene glycol (PEG) for the separation of a homogenized cell extract. After mixing, phases settle and thereby separate the different components of the cell extract on the basis of their affinity for either of the two phases, resulting in partition of the plasma membrane to the hydrophobic PEG-enriched top phase [14]. This protocol is efficient in regards to membrane protein enrichment, technical requirements, and costs. However, it is unclear if the protein yield after plasma membrane enrichment from single acute brain slices allows for liquid chromatography (LC)-MS-driven reproducible protein identification and quantification.

In this study, we demonstrate that the enrichment and identification of membrane proteins is feasible and reproducible in single acute brain slices despite the small sample mass.

## 2. Material and Methods

### 2.1. Animals

For this study, male adult Wistar rats (P40) and postnatal wild-type C57BL/6 mice (P5) were used. Day of birth was designated P0. Animals were kept at 22 ± 2 °C under a 12 h light/dark cycle with free access to water and standard diet. For rats, each cage (825 cm²) contained one or two animals, depending on the animal weight. For mice, each cage (363 cm²) contained one mother with litter. All cages were provided with bedding and nesting material. All animal-related procedures were conducted in accordance with the local ethical guidelines and the German federal animal welfare law (approval number 74.02-kau).

### 2.2. Tissue Preparation

For dissection of the rat cerebellum, adult rats were euthanized and transcardially perfused with 250 mL sodium chloride solution (0.9%). The cerebellum was dissected, weighed, and shock frozen in liquid nitrogen. Tissue was stored at −80 °C until further use.

For preparation of murine brain slices, postnatal C57BL/6 mice (P5) were decapitated and brains were quickly dissected. The tissue was embedded in 4% agarose and cut in sagittal orientation with a McIllwain tissue chopper (Ted Pella, Redding, CA, USA) into 350 µm thick slices as described in [4]. The cutting planes of all slices were documented and slices were weighed before shock freezing in cryovials in liquid nitrogen. Slices were stored at −80 °C until further use. All slices used in this study originated from the same cutting plane.

### 2.3. Sample Preparation for Whole Protein Analysis

For homogenization of tissue samples, the following solutions were added to the frozen samples: lysis buffer (9 µL/µg sample, 7 M urea, 2 M thiourea, 65 mM CHAPS hydrate, 70 mM dithiothreitol), 15% ampholytes (40%, Fluka, 39878), protease inhibitor cocktail (cOmplete™, Roche 11836153001, 0.4 µL/µg sample), PepstatinA (0.1 µL/µg sample, 0.1 mg/mL, solved in ethanol), and phenylmethanesulfonyl fluoride (0.1 µL/µg sample, 0.1 M, solved in ethanol). Samples were thawed, shock frozen, rethawed, and homogenized with a hand homogenizer (Wheaton potter and mortar, 2 mL, neolab). Afterwards, samples underwent the following circle five times: 20 s vortexing, 20 s ultrasonic bath, 20 s slewing. By then, the samples should have changed from yellow to transparent. Samples were shock frozen again, rethawed, vortexed for 30 s, and stirred on ice water for 15 min. After vortexing for another 30 s, samples were centrifuged at 17,860× *g* for 20 min at 4 °C (OptimaTM TLX, rotor TLA 110, Beckman, Brea, CA, USA). Pellet was discarded and the supernatant was stored at −80 °C until further use.

### 2.4. Plasma Membrane Enrichment

Plasma membrane protein enrichment was performed in accordance with [13]. In brief, an aqueous polymer two-phase system containing polyethylene glycol, dextrane, and Tris (tris(hydroxymethyl)aminomethane) was used for plasma membrane protein enrichment. After thawing, brain tissue was added to the two-phase system and homogenized with a homogenizer (Wheaton potter and mortar, 10 mL, neolab) and by sonification. Afterwards, phase separation was accelerated by centrifugation for 5 min at 1089× *g* and the resulting top phase was transferred to a fresh bottom phase. To enhance protein yield, the bottom phase was mixed with new top phase, then both phase systems were thoroughly mixed and again separated by centrifugation. These steps were conducted eight times in total. The top phases G and F were pooled. The resulting top phases were diluted 2:1 with 1 M KCl and 15 mM Tris (pH 7.4) and the membrane fraction was sedimented at 233,000× *g* for 1 h at 4 °C. After washing (twice with 1 M KCl/15 mM Tris (pH 7.4), thrice with 0.2 M Na_2_CO_3_), pellets were solved in lysis buffer (7 M urea, 2 M thiourea, 32.5 mM CHAPS hydrate, 5 mM dithiothreitol).

### 2.5. Measurement of Protein Concentration

For measuring protein concentrations, 4 µL of sample (in lysis buffer, see above), protein assay standard for calibration curve (Thermo Scientific, 23208, prediluted 1:5 in lysis buffer, Waltham, MA, USA), or albumin standard as a control (Thermo Scientific, 23210, prediluted 1:5 in lysis buffer) were mixed with 60 µL Pierce 660 nm protein assay reagent (Thermo Scientific, 22660). After incubation for 1 min shaking and 5 min without movement in the dark at room temperature, absorbance at 660 nm was measured in cuvettes for small volumes (Eppendorf Uvette 50–2000 µL) in a UV spectrophotometer (Ultrospec 1100pro, Amersham Bioscience, expanded by Ultrospec adapter, Amersham, UK). The calibration curve was prepared for a protein range of 0.025–0.4 µg/µL. All samples were measured in triplicates. Independent controls (0.08 µg/µL, 0.16 µg/µL, and 0.35 µg/µL albumin standard) were measured repeatedly.

### 2.6. Two-Dimensional (2D) Gel Electrophoresis

Two-dimensional gel electrophoresis was performed as previously described [15,16]. In brief, for the first dimension, the samples were diluted with rehydration buffer (6 M urea, 2 M thiurea, 32.5 mM CHAPS hydrate, 16.2 mM dithiothreitol (DTT), 2.5% ampholytes (Biochemika, 39878)). A protein mass of 8 µg in 125 µL buffer was added to Immobiline DryStrips (pH 3-10NL, 7 cm, GE Healthcare 17-6001-12). After active rehydration at 20 °C for 12 h, isoelectric focusing was performed in a Protean IEF Cell (Biorad) as follows: linear voltage rise to 300 V for 30 min, hold at 300 V for 30 min, slow voltage rise to 1000 V in 30 min, linear voltage rise to 5000 V in 90 min, hold at 5000 V for 8000 Vh.

Afterwards, stripes were rehydrated in equilibration buffer (4.4 M urea, 50.5 mM sodium dodecyl sulfate (SDS), 25 Vol% glycerol, 2.4 Vol% Tris-HCl buffer pH 8.8) containing 10 mg/mL DTT for 45 min and another 45 min in equilibration buffer with 40 mg/mL iodacetamide. Rehydrated strips were placed on precast stain-free electrophoresis gels (Mini Protean Stain free Gels 12%, BioRad, 4568041), marker (Full Range Rainbow Marker, GE Healthcare, RNP800E) was added, and stripes were overlayed with agarose solution (1% agarose, 30% glycerol, 3.4% separation gel buffer (1.5 M Tris, 14 mM SDS, pH 8.8), 55.5 mM SDS) to improve protein transfer from strip to gel. An electrophoresis chamber (Mini Protean Tetra Cell, Biorad) was filled with running buffer (TGS buffer, Biorad 161-0732) and electrophoresis was performed for 150–180 min at 100 V.

### 2.7. Silver Staining of 2D Gels

Following electrophoresis, gels were fixated in fixation solution (50% ethanol, 5% acetic acid) for 30 min. Gels were washed twice for 20 min in 50% ethanol and twice in ultrapure water for 5 min. Gels were bathed in sodium thiosulfate solution (2 mg/mL) for 1 min and washed in ultrapure water for 1 min. Afterwards, gels were incubated in silver nitrate solution (1.5 mg/mL) for 20 min and were washed for 1 min in ultrapure water. Then, gels were bathed in developer solution (0.04% formaldehyde 37%, 20 mg/mL sodium carbonate for approx. 1–10 min) until spots were detectable and reaction was stopped with 5% acetic acid. After washing in ultrapure water, gels were digitized with a ProXima 2850 imaging system.

### 2.8. In-Solution Digestion of Proteins

Samples were reduced with 10 mM DTT, subsequently sonicated for 10 min using a bath sonicator, and loaded onto Microcon YM-30-filter devices (Millipore) to perform filter-aided sample preparation (FASP) according to [17]. The processing steps for detergent removal, alkylation, buffer exchange, and protein digestion comprised two initial washes with urea solution (UA) followed by incubation with 50 mM iodoacetamide (IAA) in UA for 20 min, two washes with UA to deplete IAA, and finally three washes with 50 mM ammonium bicarbonate (ABC), before digestion with trypsin was performed at an enzyme-to-protein ratio of 1:25 in 40 µL of 50 mM ABC at 37 °C for 16 h. Peptides were collected by centrifugation and fresh trypsin solution was added onto the filter for a second digestion for 2 h. After centrifugation, the combined digests were acidified with trifluoroacetic acid (final concentration 0.25%), concentrated by use of a centrifugal evaporator and diluted to a final volume of 20 µL with a solution containing 2% acetonitrile and 0.1% formic acid (FA) in water. Peptide concentration was measured using the Qubit protein assay (Thermo Fisher Scientific, Waltham, MA, USA).

### 2.9. Analysis by nanoLC-HDMS^E^

Liquid chromatography-mass spectrometry analyses were carried out using a nanoAcquity UPLC system (Waters, Manchester, UK) coupled to a Waters Synapt G2-S mass spectrometer as described before by [18]. Mobile phase A contained 0.1% FA in water, and mobile phase B contained 0.1% FA in acetonitrile. Peptide samples corresponding to approximately 200 ng of digested protein were trapped and desalted using a precolumn (nanoAcquity UPLC Symmetry C18, 5 µm, 180 µm × 20 mm, Waters) at a flow rate of 10 µL/min for 4 min with 99.9% A. Peptides were separated on an analytical column (ACQUITY UPLC HSS T3, 1.8 µm, 75 µm × 250 mm, Waters) at a flow rate of 300 nL/min using a gradient from 3% to 32% B over 120 min for mouse samples and a gradient from 3% to 35% B over 90 min for rat samples. The column temperature was maintained at 35 °C. The SYNAPT G2-S instrument was operated in data-independent mode with ion-mobility separation as an additional dimension of separation (referred to as HDMS^E^). By executing alternate scans at low and elevated collision energy (CE) of each 0.6 s, information on precursor and fragment ions, respectively, was acquired. In low-energy MS mode, acquisitions were performed at a constant CE of 4 eV, whereas drift time-dependent CE settings [19] were applied in elevated-energy MS mode. As a reference compound, 100 fmol/μL [Glu1]-fibrinopeptide B was delivered at 500 nL/min to the reference sprayer of the NanoLockSpray source. Lock spray was acquired once every 30 s for a 1 s period. Samples were measured once without technical replication.

### 2.10. NanoLC-HDMS^E^ Data Processing, Protein Identification, and Quantification

Progenesis QI for Proteomics version 2.0 and 4.1 (Nonlinear Dynamics, Newcastle upon Tyne, UK) was used for raw data processing, protein identification, and label-free quantification of HDMS^E^ data from rat and mouse samples, respectively. Alignment was performed to compensate for between-run variation in the LC separation. Peak picking parameters included (i) sensitivity set to automatic, and (ii) a maximum ion charge of +4. Peptide and protein identifications were obtained by searching against databases containing 29,799 protein sequences of the *Rattus norvegicus* proteome (UniProt release 2017_03) and 16,970 reviewed protein sequences from *Mus musculus* (UniProt release 2018_04), respectively. Precursor and fragment ion mass tolerances were automatically determined. Two missing cleavage sites were allowed, oxidation of methionine residues was considered as variable modification, and carbamidomethylation of cysteines as fixed modification. The false discovery rate was set to 1%. Peptides were required to be identified by at least three fragment ions and proteins by at least six fragment ions and two peptides. Subsequently, peptide ion data were filtered to retain only peptide ions that met the following criteria: (1) identified at least two times within the dataset (only applied to the mouse data set), (2) ion score greater than or equal to 5.4 and 5.6 for mouse and rat data, respectively, (3) mass error below 13.0 ppm, (4) at least six amino acid residues in length. Only proteins identified by at least two unique peptides were included in the quantitative analysis of the mouse data set. Proteins were quantified by the Hi3 method [20], which uses the sum of signal intensities of the three most intense tryptic peptides of any protein. To estimate the final rate of false peptide identifications, the search was repeated using a shuffled target-decoy database applying identical peptide filtering criteria. Comparing the number of decoy peptides to those identified with the target sequences resulted in a false positive rate of 0.08%. Moreover, the search did not result in any protein identification based on more than one peptide.

The subcellular locations of identified proteins were assigned to their accession numbers using the Uniprot database. For exact reproduction of the analysis of the rat cerebellum [13], information on the subcellular localization of proteins was additionally extracted from the database Genecards.

### 2.11. Statistical Analysis

Data organization was performed in spreadsheet applications. Statistical analysis was done in SPSS25. ANOVA testing and a Mann–Whitney test were applied to determine sample variances and differences.

## 3. Results

### 3.1. Reproducibility of Plasma Membrane Enrichment Protocol

**Figure 1:**
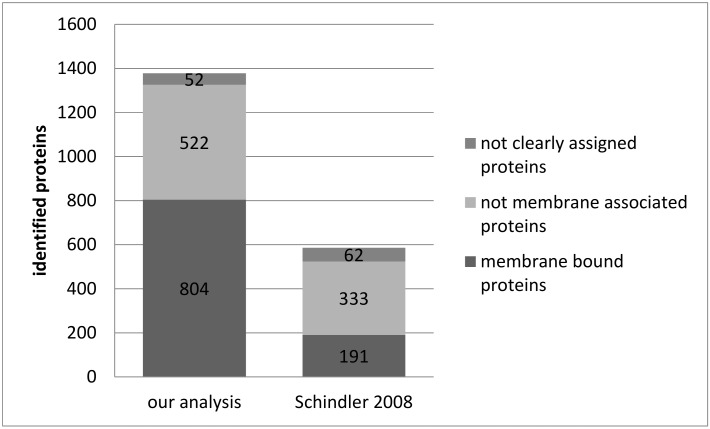
The number of protein identifications in plasma-membrane-enriched samples of rat cerebellum. Plasma membrane proteins were enriched in samples of adult rat cerebellum. Proteins were identified with LC-MS. Subcellular protein localization was assigned with aid of the database Genecards.

To ensure technical reproducibility of plasma membrane separation and identification of the Schindler protocol [13], the whole procedure was performed using the cerebella of two adult Wistar rats. By means of LC-MS, 1378 proteins were identified by at least one unique peptide (Table S1). The subcellular localizations of these proteins were assigned using the database Genecards (Figure 1). Of the total 1378 proteins, 804 (58%) were assigned to membranes, and 522 proteins (38%) were not assigned to membranes. For 52 proteins (4%), no information on the subcellular localization was available. For comparison, Schindler et al. [13] identified 586 proteins, of which 191 (33%) were assigned to membranes by the use of Genecards. In their original article, Schindler et al. defined a list of selected plasma membrane proteins with neurobiological relevance. Almost all of these proteins were also found in our analysis, as demonstrated in Table 1. Six proteins (Dihydropyridine-sensitive L-type, calcium channel alpha-2/delta subunits, Potassium voltage-gated channel subfamily C member 3, Sodium channel protein type 1 subunit alpha, Sodium bicarbonate cotransporter 3, Electrogenic sodium bicarbonate cotransporter 1, Plasma membrane calcium-transporting ATPase 3) reported in [13] were not shown in Table 1 because they were associated with a protein group and not confirmed by unique peptides in our analysis. In addition, typical membrane proteins that were identified here, but were not described in [13], are introduced in the following. The adhesion G protein-coupled receptor L3 (ADGRL3), which has functions in cell–cell adhesion as well as neuron guidance and is necessary for the development of glutamatergic synapses in the cortex, was identified in different samples. A further example is the ciliary neurotrophic factor receptor subunit alpha (CNTFR), which binds the neurotrophin CNTF. CNTF promotes neurotransmitter synthesis and neurite outgrowth in certain neural populations. Beyond this, four gamma-aminobutyric acid (GABA) and glycine transporter proteins were identified. These are sodium- and chloride-dependent GABA transporter 1, sodium- and chloride-dependent GABA transporter 3, sodium- and chloride-dependent glycine transporter 1, and sodium- and chloride-dependent glycine transporter 2. Again, these four transmembrane proteins were not identified by Schindler et al. [13].

Excerpt of identified membrane proteins from Schindler et al. [13]. The Swiss-Prot primary accession number, protein names, the number of transmembrane helices (TMH), and the number of identified peptides per protein in Schindler’s analysis (pep) [13] and in our analysis (A/B: sample A/B) are listed. Seven proteins that were identified in Schindler et al. have not been identified in our samples and, therefore, are not shown in this table. Six proteins that were associated with a protein group but not confirmed by unique peptides in our analysis were also not included.

### 3.2. Protein Analysis in Single Acute Slices

**Figure 2:**
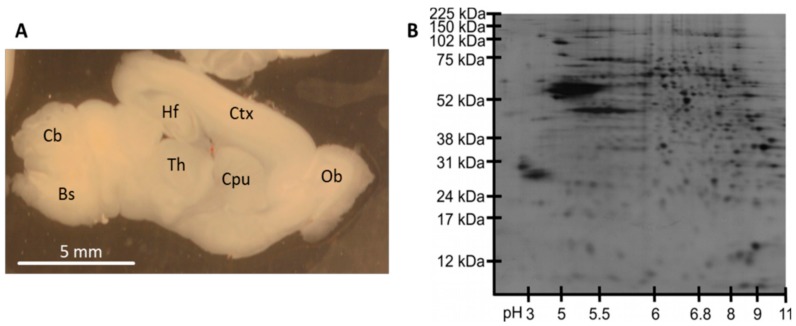
Protein analysis in single acute slices. (**A**) Acute slices were prepared from 5-day-old C57BL/6 mice. Sagittally cut slices contained cortex (Ctx), hippocampal formation (Hf), thalamus (Th), caudate–putamen complex (Cpu), olfactory bulb (OB), cerebellum (Cb), and brain stem (Bs). (**B**) Two-dimensional protein separation of the whole-protein fraction of one single acute slice demonstrated abundance of various proteins over a large protein weight and pI range.

For all subsequent experiments, acute slices were prepared from 5-day-old C57BL/6 mice (Figure 2A). As our group strives for analyses of the proteome of organotypic slices during culture, slices for this study were prepared in the very same way as slices for organotypic culturing. Slices were cut sagittally at a thickness of 350 µm and weighed between 9 and 19 mg. To ensure that the protein mass of one single slice is sufficient for protein-based analysis approaches, we performed 2D gel electrophoresis of the whole protein fraction of single slices (Figure 2B). It turned out that the protein mass of a single slice is sufficient to perform a 2D gel electrophoresis. The distribution of protein spots in the resulting gels demonstrated the availability of proteins over the complete isoelectric point (pI) range from 3 to 11 as well as the protein size range from 10 kDa to 225 kDa.

**Figure 3:**
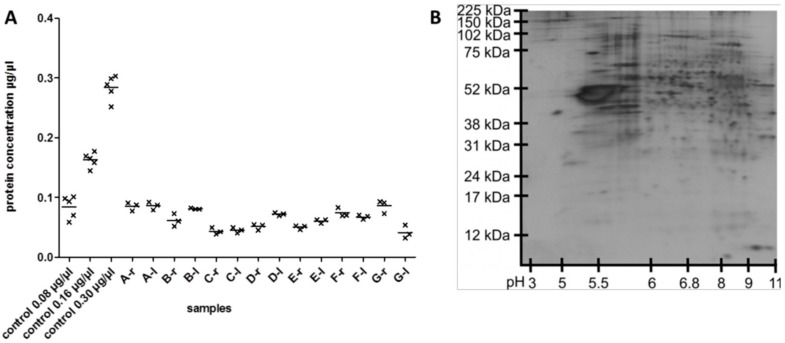
Protein content after membrane protein enrichment in single acute slices. (**A**) Protein concentrations after membrane protein enrichment in single slice samples. Samples were termed after the animal (letter) and originate from right or left hemispheres (r or l). Protein concentration was determined three times per sample. (**B**) Two-dimensional protein separation after membrane protein enrichment of one single acute slice demonstrated abundance of various proteins over a large protein weight and pI range.

Single acute slices were then processed for plasma membrane enrichment following the protocol of Schindler et al. [13]. Performing this protocol with single slice samples proved to be challenging because pellets after ultracentrifugation of the enriched membrane fraction were not visible due to their small size. Furthermore, protein concentration measurements had to be adjusted to small sample sizes and low protein concentrations. As illustrated in Figure 3A, protein concentrations of processed samples ranged between 0.04 and 0.09 µg/µL, corresponding to an available protein amount of about 3–6 µg for subsequent MS analyses. Accuracy of the protein concentration measurements was ensured by determining the protein concentration of every sample three times. The standard error of the mean of protein concentration measurements from the same samples was considerably small and did not exceed 0.007. For visualization of protein content of the processed slice samples, 2D gel electrophoresis was performed (Figure 3B). Protein spots were distributed over the complete analyzed pI and protein size range. However, the 2D gel also showed that samples after plasma membrane enrichment contained a significant amount of proteins not intrinsic to membranes because hydrophobic membrane proteins are typically not resolved by 2D gel electrophoresis.

### 3.3. Plasma Membrane Protein Separation in Single Acute Slices

For evaluation of the reproducibility of the plasma membrane separation protocol in single slices, acute slices from seven animals were chosen for MS analysis. Per animal, one slice per hemisphere from the same cutting plane was processed. By means of LS-MS, an average of 8000 peptides was identified. A total of 1161 proteins was identified by at least two peptides (Table S2), while 248 proteins were identified by only one peptide and, therefore, were excluded from analysis due to insufficient specificity of identification and quantification. One sample showed considerably lower protein concentration (sample G-l in Figure 3A) and peptide as well as protein identifications. This sample and the corresponding sample from the same animal were excluded from analysis.

**Figure 4:**
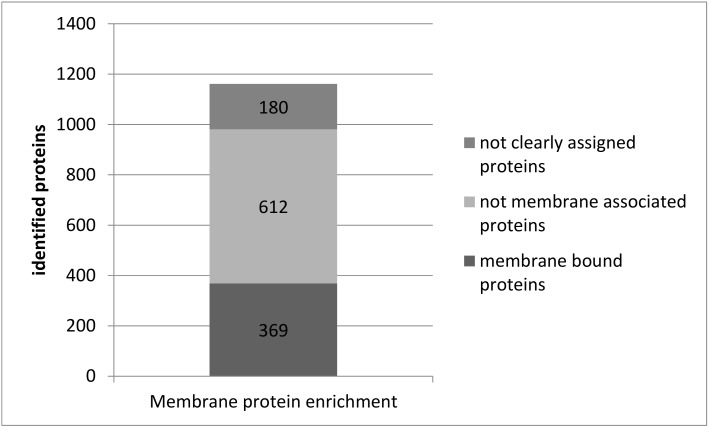
The number of protein identifications in plasma-membrane-enriched samples of single acute slices. Plasma membrane proteins were enriched in samples of single murine acute slices. Proteins were identified with LC-MS. Subcellular protein localization was assigned with aid of the database Uniprot.

Protein localizations were assessed with the aid of Uniprot. A total of 369 proteins (32%) were assigned to membranes, 612 proteins (53%) were assigned to other subcellular compartments, while no localization information was available in Uniprot for 180 proteins (16%) (Figure 4).

For evaluation of constancy of abundance measurements in the 12 samples, the difference between the minimal and maximal mean abundance per animal was calculated for each protein and expressed as fold-change (Table S2). Furthermore, ANOVA testing was performed to analyze if abundances of all samples were significantly different. For 164 proteins, the fold change between maximal and minimal abundance was larger than 2. Seventy-eight of these proteins also had a *p*-value < 0.05 and therefore were supposed to be significantly different. Regarding all proteins, 762 out of 1161 showed a *p*-value > 0.05 after ANOVA and therefore showed no significant variability (Table S2).

**Figure 5:**
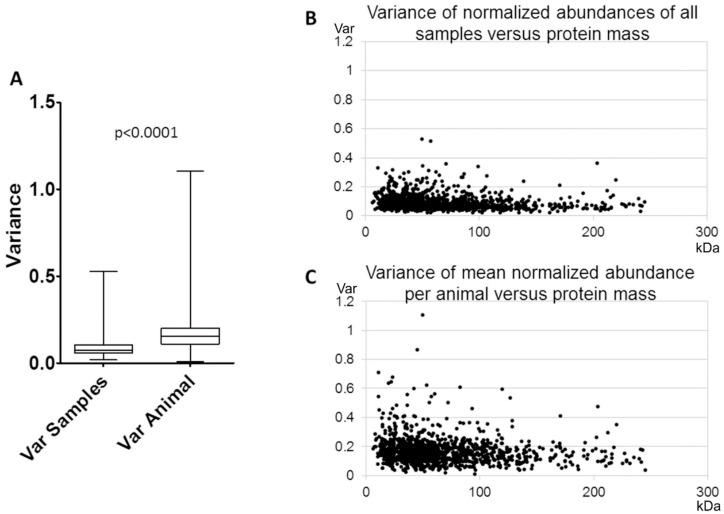
Variance of normalized protein abundance in single acute slices. (**A**) For each identified protein, the variance of normalized abundances for all samples was calculated (Var Samples) as well as the variance of the mean protein abundances per animal (Var Animal). (**B**) The variances of normalized abundances of all samples were plotted against the weight of the respective protein. No correlation was found (r = −0.14744). (**C**) The variances of the mean normalized abundances per animal were plotted against the weight of the respective protein. No correlation was found (r = 0.13419).

We hypothesized that the amounts of identified proteins were comparable in all samples since they all originated from comparable animals and the same cutting planes. So, the variance of abundances from all 12 samples was calculated for every identified protein. Variances of single slice samples were 0.091 ± 0.053 on average. Furthermore, we assumed that, due to interindividual differences between the animals, slices from different animals would vary more in their protein amounts than slices from the same animal. Hence, the mean abundance from both samples of an animal was determined and then the variances of these means for every identified protein were calculated. The overall average variance of single animal protein amounts was 0.168 ± 0.095 on average. This analysis demonstrated that variance of the samples from individual animals was significantly higher than variance of all samples (*p* < 0.0001, Figure 5A).

Furthermore, variances were plotted against the protein weight of the respective proteins. The distribution of variances between samples showed a lower variability (Figure 5B) than between (Figure 5C) animals. No correlation was found between variances of single samples and protein mass nor between variances of animals and protein mass (Pearson correlation test, r = −0.14744, respectively r = 0.13419, Figure 5B).

The identified proteins of the membrane protein enrichment procedure in the rat cerebellum were compared with the myelin proteome of Jahn et al. [21] to prove coincidence with proteins of the myelin proteome, respectively, oligodendrocyte compartment. Interestingly, we found 35 proteins in our membrane protein enrichment samples of the rat cerebellum that were also described in the myelin proteome: 14-3-3 protein epsilon, 14-3-3 protein eta, 14-3-3 protein gamma, 14-3-3 protein theta, annexin A6, calnexin, clathrin heavy chain, cofilin 2, destrin, elongation factor 2, gelsolin, glial fibrillary acidic protein, glucose-6-phosphate isomerase, glutamine synthetase, heat shock 70 kDa protein 1B, heat shock 70 kDa protein 4, macrophage migration inhibitory factor, moesin, myelin basic protein, myelin proteolipid protein, myelin-associated glycoprotein, neural cell adhesion molecule 1, neural cell adhesion molecule 1, neurofascin, neurotrimin, nucleoside diphosphate kinase A, nucleoside diphosphate kinase B, phosphoglycerate mutase 1, prohibitin, septin 4, synaptophysin, transketolase, triosephosphate isomerase, and vimentin.

## 4. Discussion

### 4.1. Reproducibility of the Plasma Membrane Enrichment Protocol

The protocol of Schindler et al. [13] was successfully reproduced with material of the cerebellum of adult Wistar rats (Figure 1, Table S1). A large number of proteins was identified by LC-MS, and more than half of them are assigned to plasma membranes in the database Genecards. In Schindler’s original analysis, only half as many proteins could be identified in the same sample tissue, and a considerably smaller amount of them were assigned to membranes. This notable increase in protein identification efficiency most certainly is due to advances in MS technology and the expanded scope of database content during the last decade. The successful reproduction of plasma membrane protein enrichment and identification is the prerequisite for applying the protocol to the small sample volume of single acute murine slices.

### 4.2. Protein Analysis in Single Acute Slices

Protein-based analyses of single slices are rarely executed due to their small sample volume. We were able to demonstrate that one single acute murine slice provides enough protein for two-dimensional gel electrophoresis. The whole protein fraction of one single slice contains a broad range of sufficiently separable protein spots, indicating the suitability of the sample format of acute slices for protein-based analyses (Figure 2).

Based on this result, plasma membrane protein enrichment was performed on single acute murine slices. We anticipated a considerable loss of protein content due to the high number of processing steps in the protocol (eightfold repetition of phase separation), but the separation of the enriched membrane protein fraction in two-dimensional gel electrophoresis demonstrated a remarkable number of distinct protein spots (Figure 3B).

Since the sample preparation for MS requires the measurement of protein concentration, the protocol for protein measurements needed to be optimized for low sample volumes. Despite the use of small volumes for protein measurement and a comparably low protein concentration range of the standard curve, measurements of protein concentrations were reproducible for all samples measured. The protein concentration of every sample was measured in triplicates, and the low variability of the resulting protein concentrations for each sample proved the applicability of our optimized protein measurement protocol (Figure 3A).

### 4.3. Membrane Proteins in Single Acute Slices

The MS-based protein identification of membrane protein-enriched single murine slice samples resulted in the identification of 1161 proteins (Table S2). By using the database Uniprot, 369 of these proteins (31.8%) were allocated to membranes (Figure 4). For comparison, the enrichment of membrane proteins in whole rat cerebellum resulted in 804 membrane protein identifications (58.3% of all identified proteins, Figure 1), a remarkably higher yield. However, comparability of both analyses is limited due to samples from different species and different brain regions. Therefore, we consider the yield of membrane proteins from single acute slices as sufficient for further analyses.

The most important prerequisite for the use of MS-based analysis approaches on single slices is reliable reproducibility of the results. For evaluation of this subject, we analyzed slices from seven mice from one litter, two slices per animal, all from the same cutting levels. Out of the 14 samples, one sample yielded insufficient protein concentrations and was excluded from further analysis. We therefore consider the probability of extensive sample loss during membrane protein enrichment as acceptable given the sophisticated enrichment protocol.

For evaluation of reproducibility of MS protein quantification, the variance of normalized abundances of all samples (sample variance) was compared with the variance of the mean normalized abundances per animal (animal variance) (Figure 5). The results show that the animal variance is considerably higher than the sample variance. We conclude that (i) slices from the same animal reliably have comparable protein abundances and that (ii) our method is sensitive enough to detect interindividual differences of protein abundances in single slice samples.

To further evaluate the constancy of protein quantifications in samples from different animals, the fold change between highest and lowest mean abundance per animal for every protein identified was calculated (Table S2). For 14.1% of all identified proteins, a fold change larger than 2 was found. ANOVA testing proved that only 78 of these proteins (6.7% of all identified proteins) showed significant variability of abundances and, therefore, can be considered to be differential. This amount of differentially expressed proteins appears to be realistic because interindividual differences of protein profiles between individuals of inbred mice of the same strain and age physiologically exist.

Taken together, our results prove that the membrane protein profile of single slice samples can be analyzed by LC-MS. The small sample volume constitutes no restriction for reproducible and reliable protein identification and quantification. Since acute brain slices can be analyzed with this method, it can be assumed that cultured organotypic slices also are a suitable sample format for MS-based analysis. This enables studies on the changes of protein profiles over the course of organotypic slices propagation as well as protein expression analyses in various lesion or intoxication models of organotypic slice cultures [22,23]. However, the application of the method is not restricted to slice preparations. Also, small dissected brain areas with minor sample weight can probably be employed for enrichment of plasma membrane proteins and MS protein identification.

The technique of plasma membrane protein enrichment and MS protein identification is now ready for specific differential analyses in neuroscientific research. One example is the investigation of differential protein abundances in models of de- and remyelination in the context of multiple sclerosis. It was shown that 35 proteins of the myelin proteome [21] were also identified by the plasma membrane enrichment approach. Both organotypic slice culture models with demyelinating lesions [24,25] and single topographic regions dissected from murine brains (corpus callosum, cerebellum, spinal cord [26,27]) are difficult to analyze due to their small sample volume and protein mass. However, by the use of our protocol presented in this study, these samples are now accessible for proteome-based investigation.

## Figures and Tables

**Table 1:** Comparison of cerebellar protein identifications.

Accession		TMH	Schindler et al.	Our Analysis	
	**Neurotransmitter Release**		**pep**	**A**	**B**
P61765	Syntaxin-binding protein 1	0	30	43	43
Q9WU70	Syntaxin-binding protein 5	1	1	4	4
P32851	Syntaxin-1A	1	6	4	4
P61265	Syntaxin-1B2	1	21	17	16
P60881	Synaptosomal-associated protein 25	0	19	19	19
	**Neurotransmitter Receptors**				
P19490	Glutamate receptor 1	5	3	9	9
Q63226	Glutamate receptor delta-2 subunit	3	6	28	29
P23385	Metabotropic glutamate receptor 1	8	6	23	17
Q9Z0U4	Gamma-aminobutyric acid type B receptor, subunit 1	8	3	8	6
O88871	Gamma-aminobutyric acid type B receptor, subunit 2	8	2	10	10
P62813	Gamma-aminobutyric-acid receptor alpha-1 subunit	5	5	4	4
P30191	Gamma-aminobutyric-acid receptor alpha-6 subunit	4	0	8	7
P63138	Gamma-aminobutyric-acid receptor subunit beta-2	5	5	3	3
P18506	Gamma-aminobutyric-acid receptor delta subunit	5	2	3	3
P18508	Gamma-aminobutyric-acid receptor gamma-2 subunit	5	3	0	1
	**Neurotransmitter Reuptake**				
P31662	Orphan sodium- and chloride-dependent neurotransmitter transporter NTT4	11	12	5	5
P23978	Sodium- and chloride-dependent GABA transporter 1	12	5	5	7
P31647	Sodium- and chloride-dependent GABA transporter 3	11	8	6	5
P28572	Sodium- and chloride-dependent glycine transporter 1	12	4	4	4
P24942	Excitatory amino acid transporter 1	10	14	8	7
P31596	Excitatory amino acid transporter 2	11	12	9	7
O35921	Excitatory amino acid transporter 4	8	7	7	6
	**Ion Channels**				
Q9Z2L0	Voltage-dependent anion-selective channel protein 1	0	9	12	11
P10499	Potassium voltage-gated channel subfamily A member 1	6	4	1	1
P25122	Potassium voltage-gated channel subfamily C member 1	7	4	3	3
P04775	Sodium channel protein type 2 subunit alpha	24	8	3	3
Q00954	Sodium channel beta-1 subunit	2	1	2	2
P54900	Sodium channel beta-2 subunit	2	5	4	5
	**Transporters**				
Q9JHZ9	System N amino acid transporter 1	10	2	0	1
P11167	Solute carrier family 2, facilitated glucose transporter member 1	11	3	3	2
Q8VII6	Choline transporter-like protein 1	10	1	1	0
Q63016	Large neutral amino acids transporter small subunit 1	14	3	5	5
Q63633	Solute carrier family 12 member 5	12	12	17	15
P11505	Plasma membrane calcium-transporting ATPase 1	9	23	13	19
P11506	Plasma membrane calcium-transporting ATPase 2	9	36	2	3
Q64542	Plasma membrane calcium-transporting ATPase 4	11	13	10	9
P06685	Sodium/potassium-transporting ATPase alpha-1 chain	8	42	29	28
P06686	Sodium/potassium-transporting ATPase alpha-2 chain	8	48	28	28
P06687	Sodium/potassium-transporting ATPase alpha-3 chain	8	50	32	32
P07340	Sodium/potassium-transporting ATPase subunit beta-1	1	14	15	15
P13638	Sodium/potassium-transporting ATPase subunit beta-2	1	10	8	9
Q63377	Sodium/potassium-transporting ATPase subunit beta-3	1	5	7	7
P53987	Monocarboxylate transporter 1	12	2	4	5
Q01728	Sodium/calcium exchanger 1	11	3	4	4
P48768	Sodium/calcium exchanger 2	11	11	14	15

## Supplementary Materials

The following are available online at https://www.mdpi.com/2073-4409/8/5/423/s1. Table S1: Identified proteins after membrane protein enrichment in rat cerebellum, 1378 proteins were identified. Proteins were sorted with regard to number of membrane descriptions. mem: number of words “membrane” in localization description of proteins. Table S2: Identified proteins after membrane protein enrichment in single acute murine slices, 1161 proteins were identified. Proteins were sorted by descending *p*-values of ANOVA testing for homogeneity between all samples. *p*: probability error ANOVA testing, Change: fold change between smallest and highest measured mean abundance per animal, mem: number of words “membrane” in localization description of proteins. Samples were named after the animal (letter) and origin from the right or the left hemisphere (r or l).
